# Supplementary material for: Lipid unsaturation promotes BAX and BAK pore activity during apoptosis
Source: Nat Commun. 2024 Jun 3;15:4700. doi: 10.1038/s41467-024-49067-6 (PMC11148036; doi:10.1038/s41467-024-49067-6)
Supplement: Supplementary file 1 — Supplementary Information [file 41467_2024_49067_MOESM1_ESM.pdf]

Supplementary Information

Supplementary Figure 1A

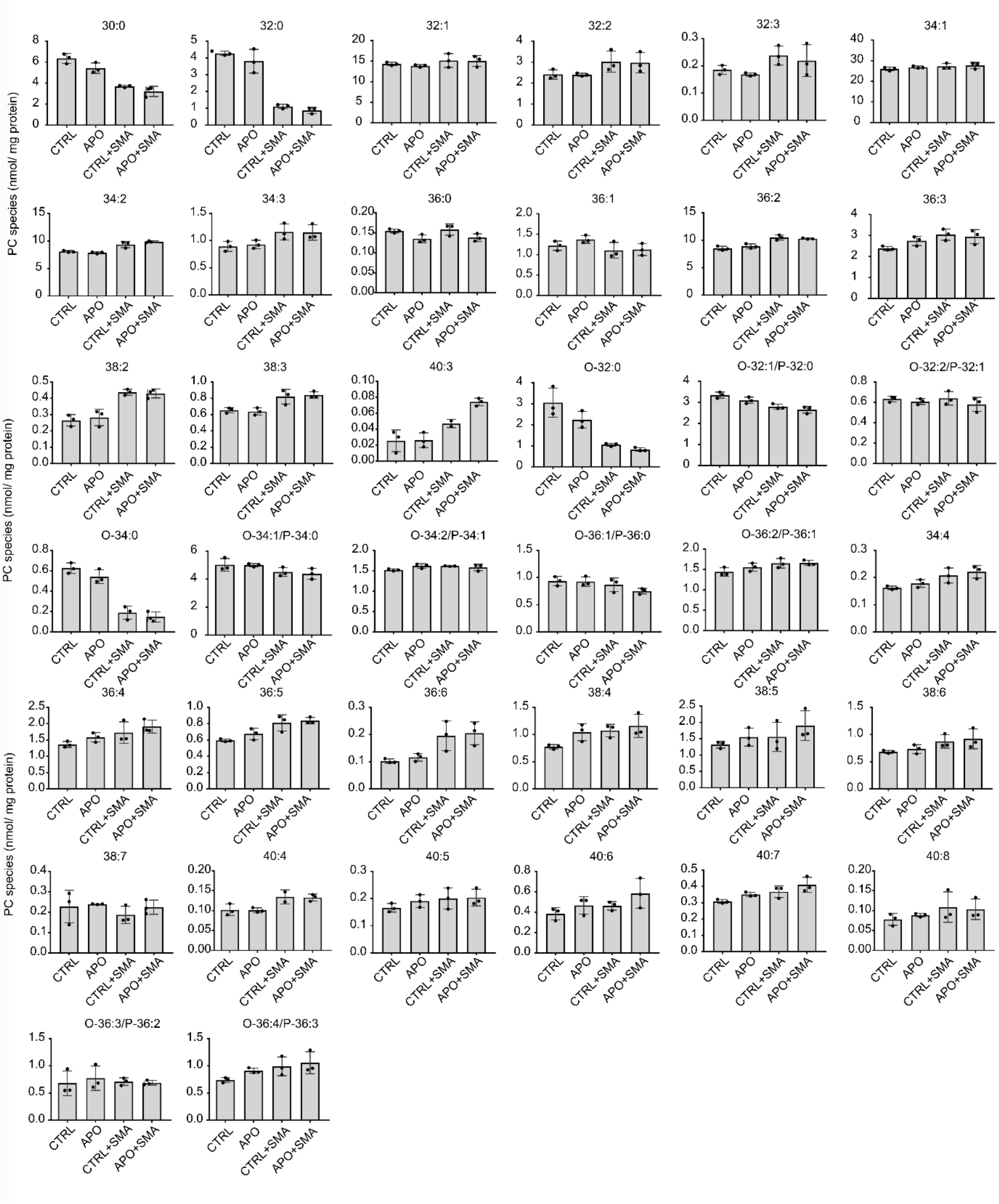

Supplementary Figure 1B

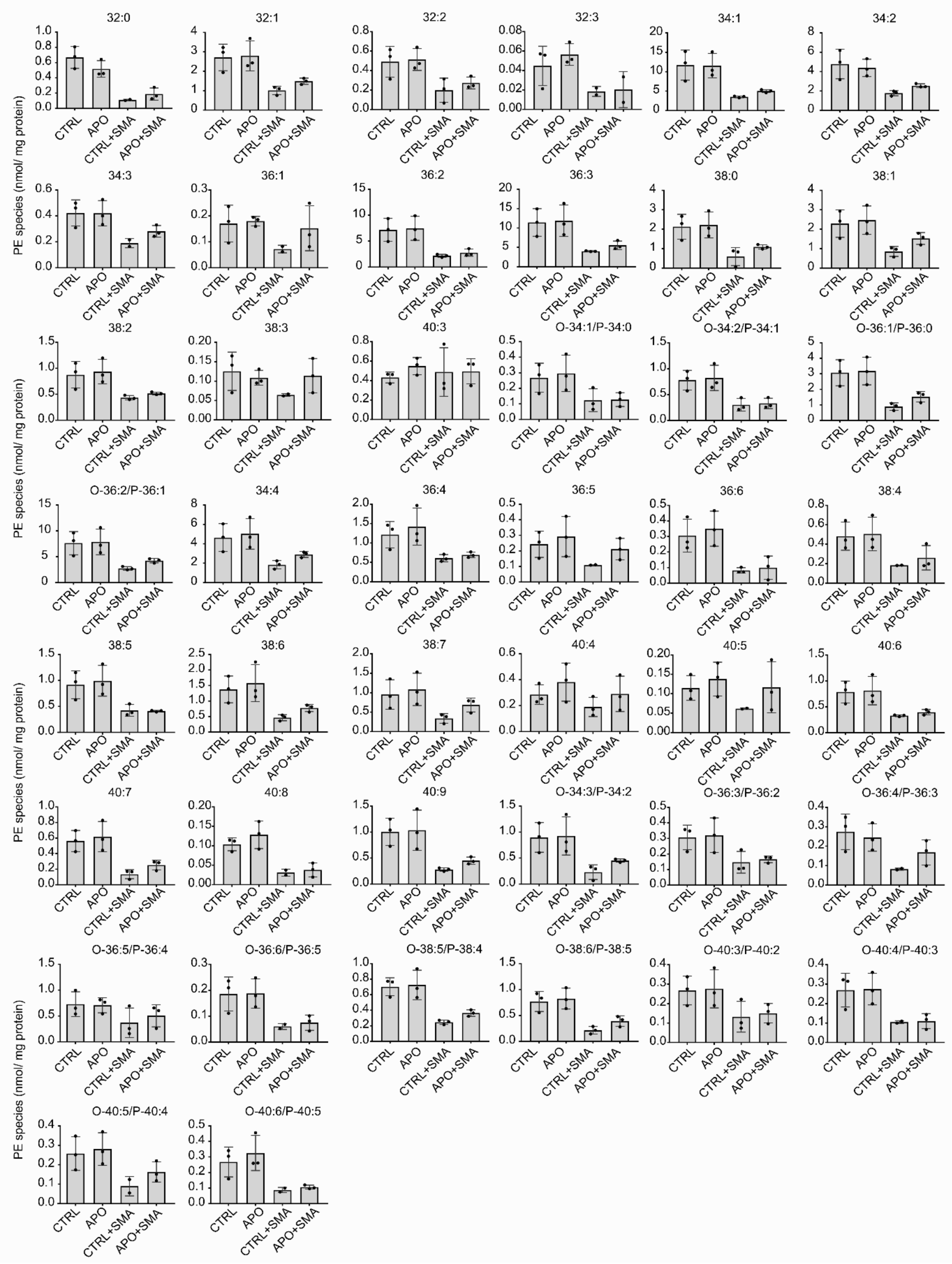

Supplementary Figure 1C

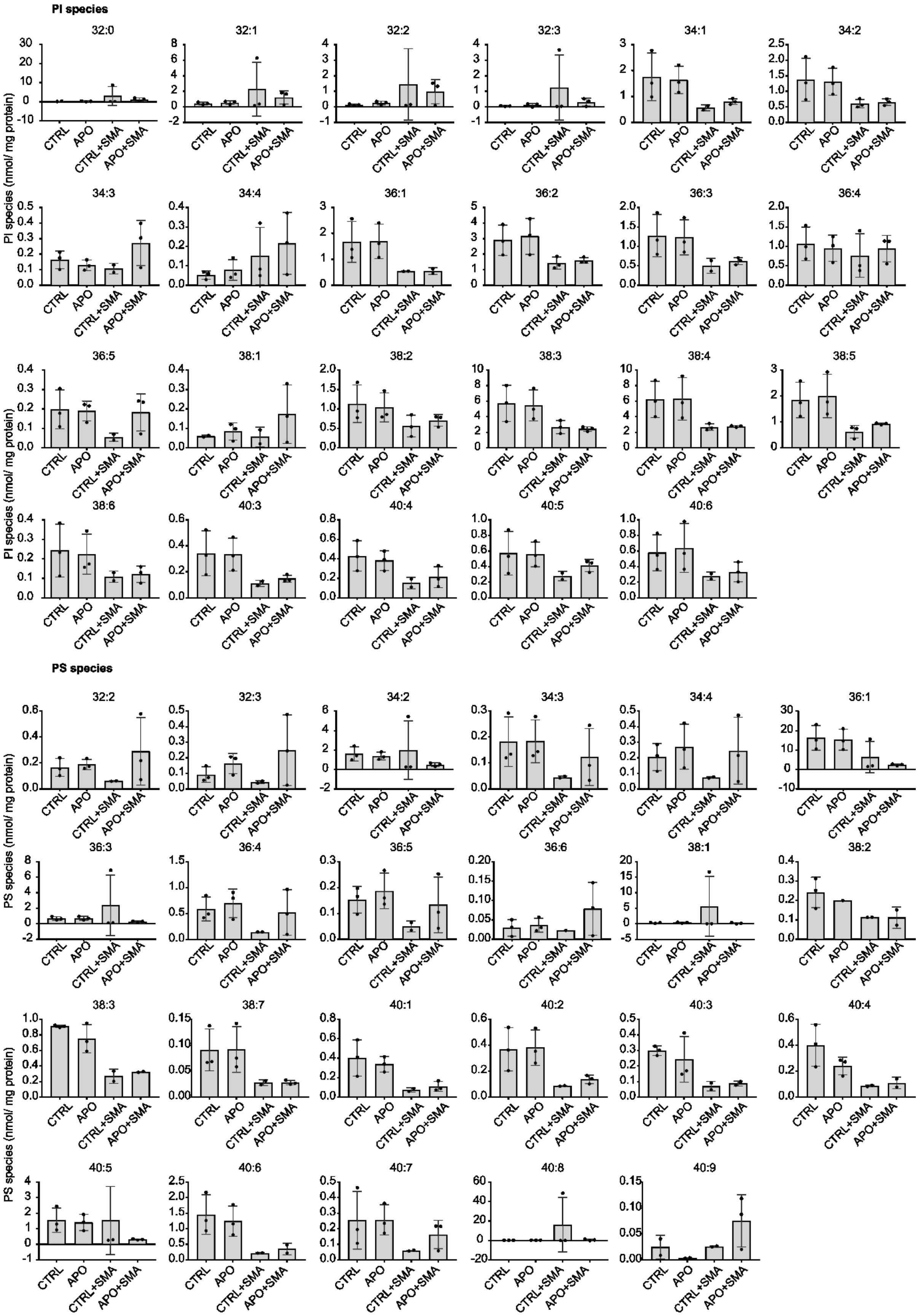

Supplementary Figure 1D

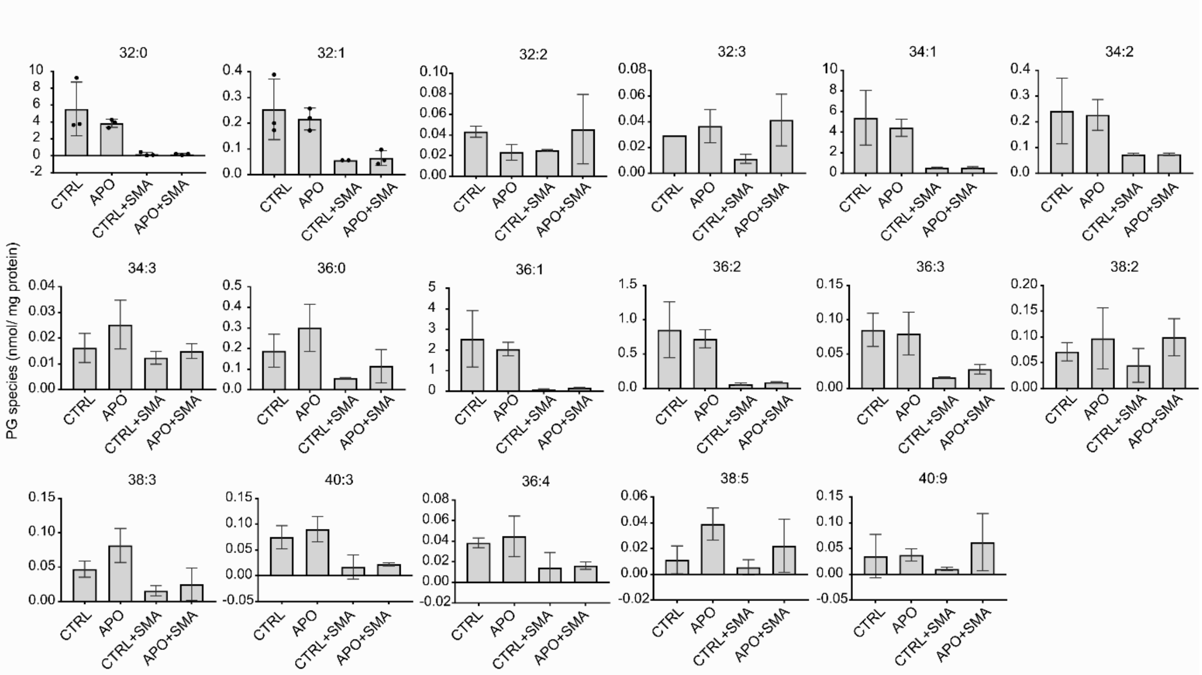

Supplementary Figure 1E

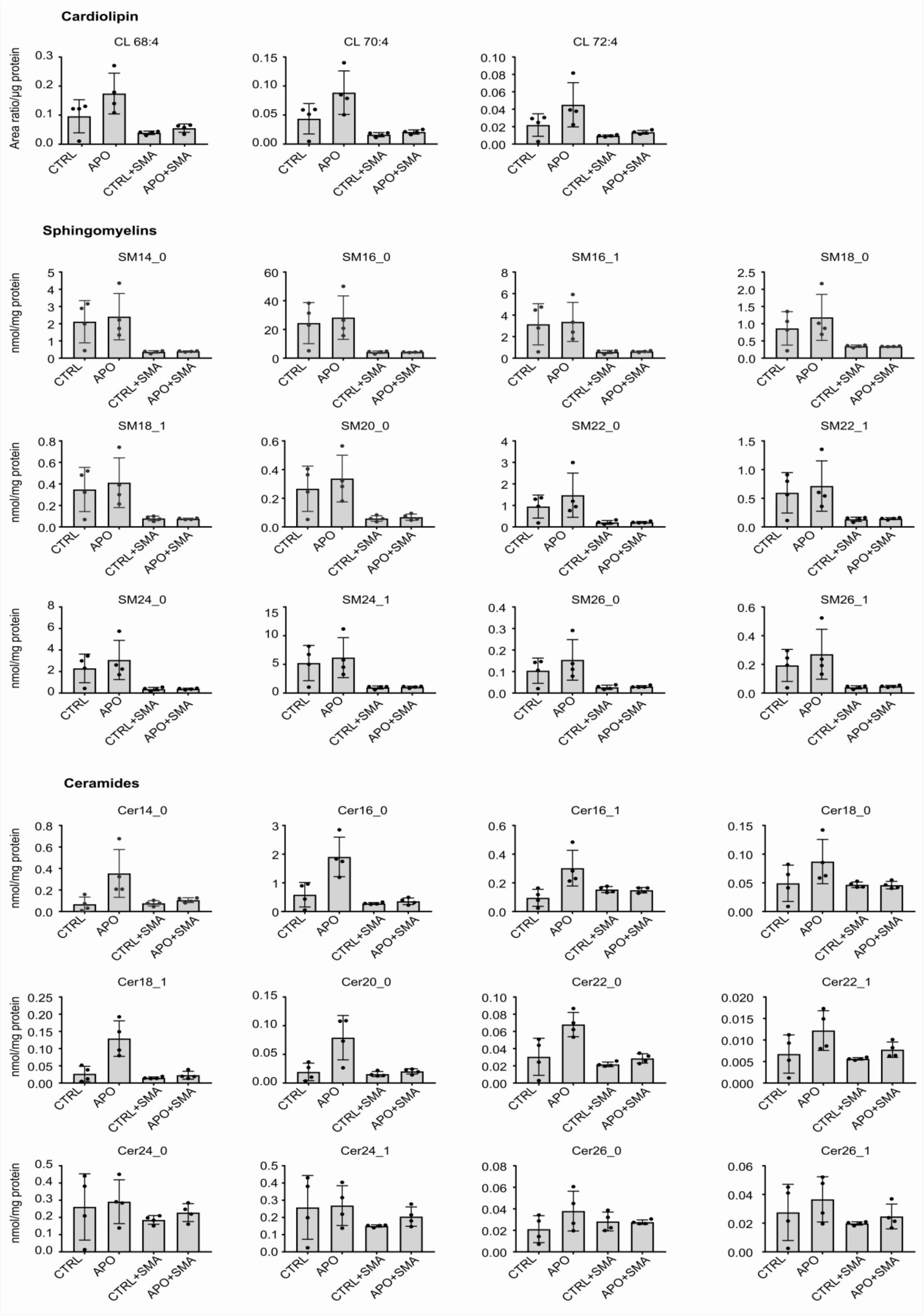

Supplementary Figure 1F

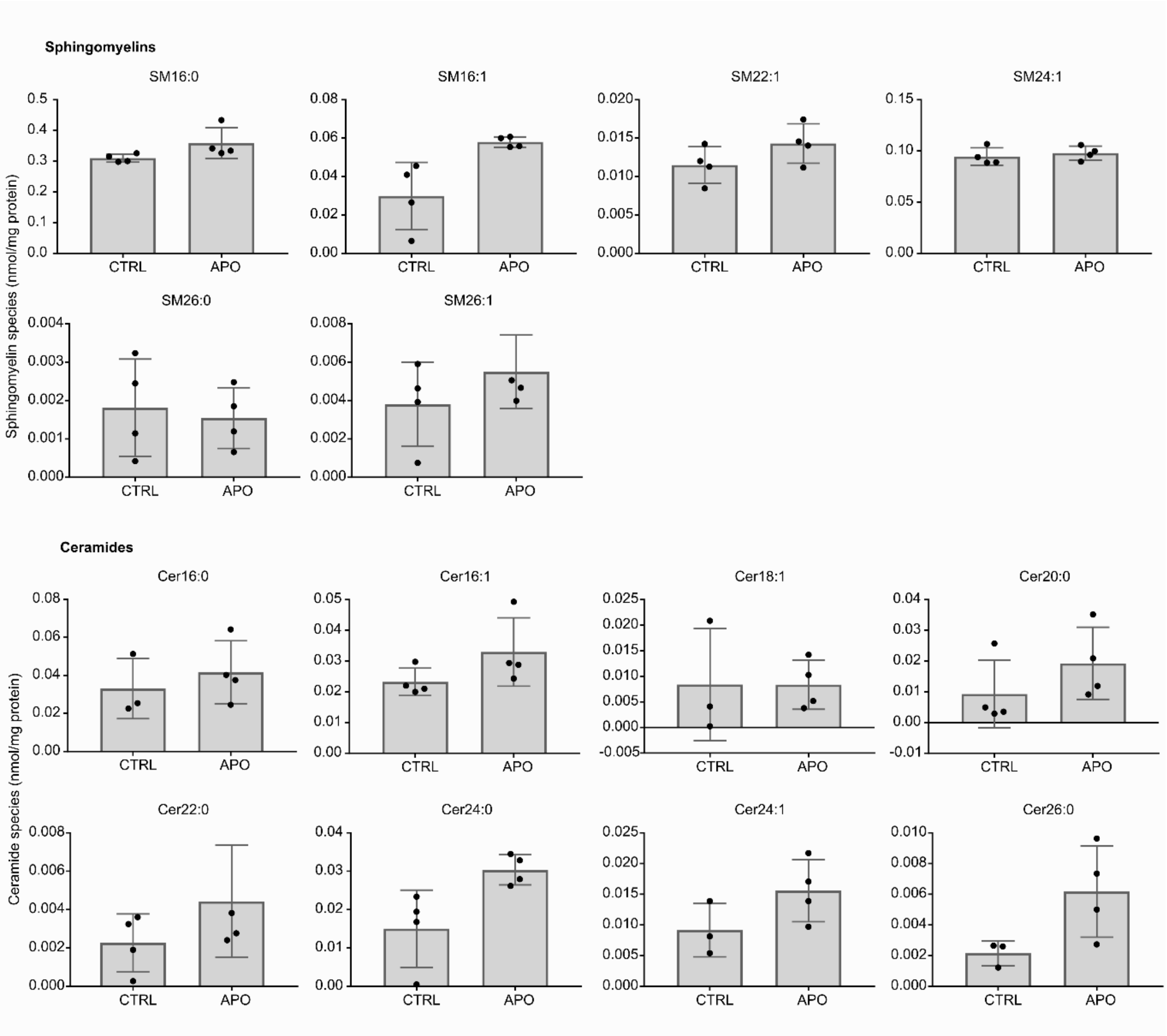

Supplementary Figure 1. Phospholipid profiles of total mitochondria extracts and mitochondrial SMALPs isolated from healthy and apoptotic WT U2OS cells.

(A) PC; Phosphatidylcholine, (B) PE; Phosphatidylethanolamine, (C) PI; Phosphatidylinositol and PS; Phosphatidylserine, (D) Phosphatidylglycerol (PG) Phosphatidic acid (PA), and (E) Cardiolipin and sphingolipids. (F) Sphingolipids profile from mEGFP-BAK SMALP. Data points represent three replicates. Error bars represent ± S.D.

Supplementary Figure 2

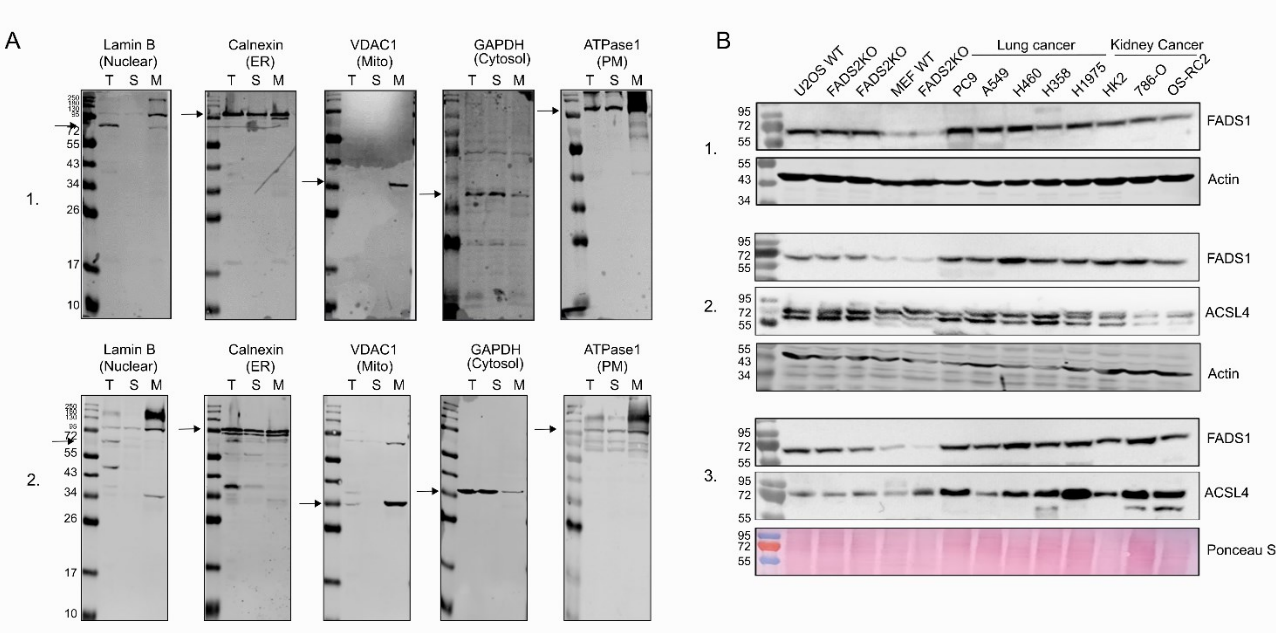

Supplementary Figure 2.

(A) Quality analysis of crude mitochondrial isolation by WB analysis. T, total; S, supernatant; M, mitochondrial pellet fraction. Repetitions numbered.

(B) FADS1 and ACSL4 expression in various cell lines used in this study, detected by WB. Repetitions numbered.

Supplementary Figure 3

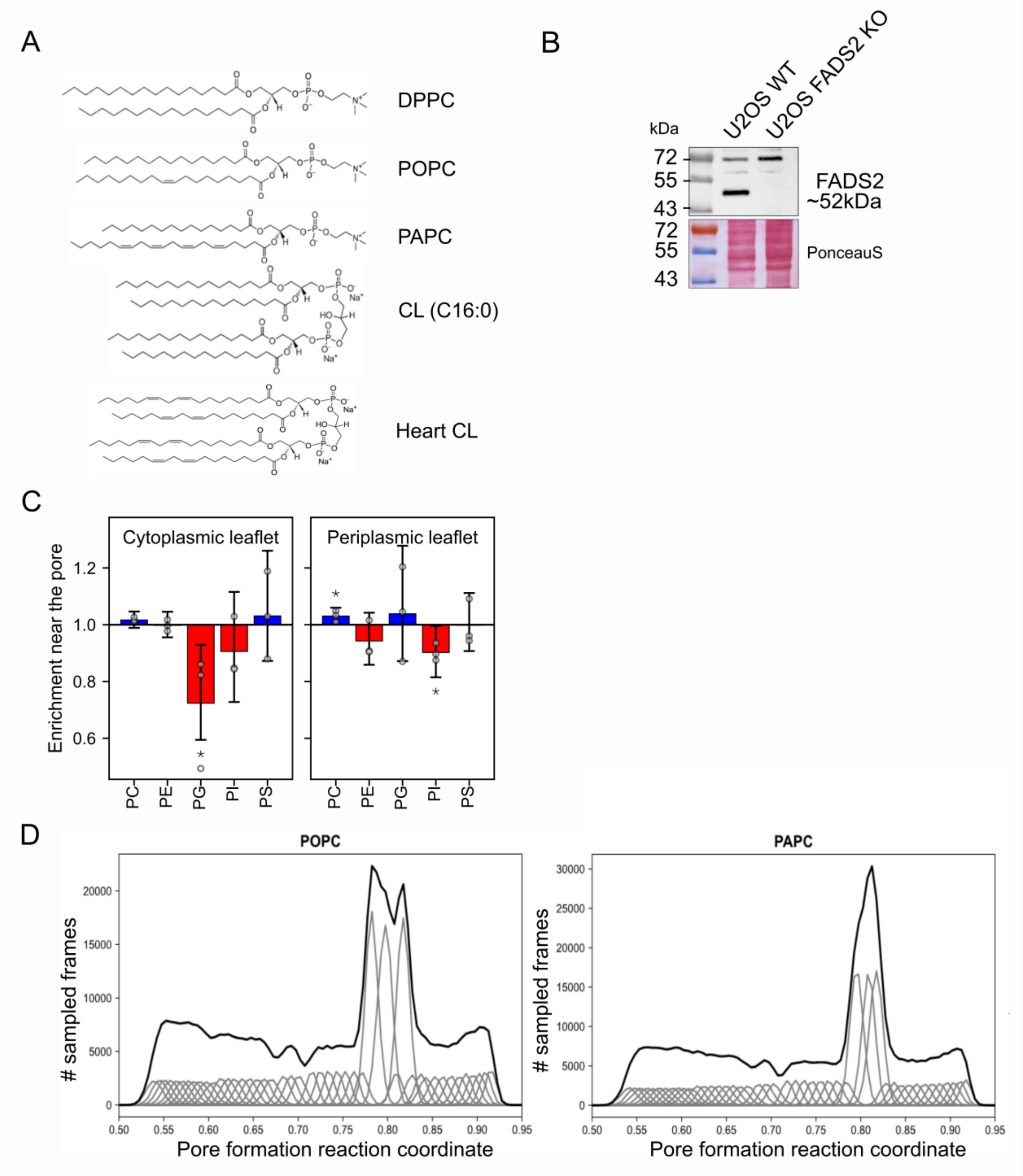

Supplementary Figure 3.

(A) Molecular structures of PC and cardiolipin species with varying unsaturation used in this study to generate LUVs and GUVs. DPPC (1,2-Dipalmitoyl-sn-glycero-3-PC), PAPC (1-Palmitoyl-2-arachidonoyl-sn-glycero-3-PC), POPC (1-palmitoyl-2-oleoyl-sn-glycero-3-PC), 16:0 Cardiolipin (1',3'-bis[1,2-dipalmitoyl-sn-glycero-3-phospho]-glycerol).

(B) Validation of FADS2 KO in U2OS WT cells by WB analysis of FADS2 expression levels.

(C) Fold change of major lipid species near a toroidal pore calculated from CG-MD simulations. For coarse-grain Martini 3 models, the lipid bilayer mimicking the MOM was configured based on lipidomic data from Figure 2. Pore formation induced by position restraints. 3 points, each the average of one of the replicates. Asterisks indicate a significant enrichment (blue bars) or depletion (red bars) with a confidence of 99%.

(D) Sampled data in the umbrella-sampling simulations of pore formation in POPC or PAPC membranes. The sampling of each of the 45 windows is histogrammed individually (gray lines) and as a total (black lines) for pore formation reaction coordinate. Sampling was done every 10 ps and histogramming was done into bins of width 0.005, yielding a minimum of 3667 (POPC) and 3786 (PAPC) samples per bin in the 0.55 to 0.90 range. Considering time correlation, each window had a minimum of 190 (POPC) or 102 (PAPC) uncorrelated data segments, with windows in the 0.55 to 0.90 range having a minimum 214 (POPC) or 252 (PAPC) uncorrelated data segments. Selected windows close to pore coordinate 0.8 were extended to compensate for large autocorrelation times, presumably due to proximity to a transition point and relatively slow exchanges over it.

Supplementary Figure 4

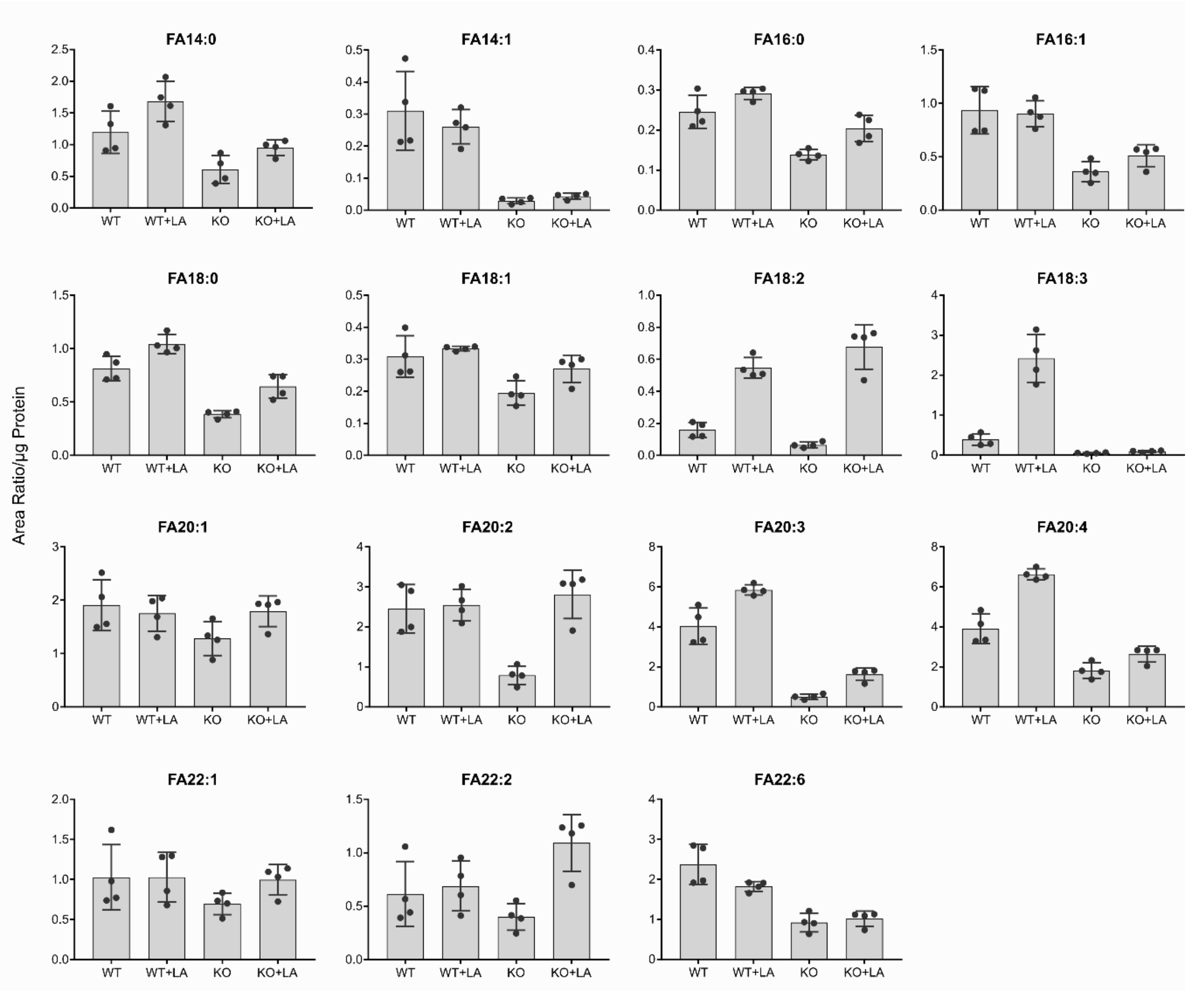

Supplementary Figure 4.

Total fatty acid profile of isolated mitochondria from WT U2OS and FADS2 KO cells treated or not with Linoleic acid prior to mitochondria isolation. Data points represent four replicates. Error bars represent ± S.D.

Supplementary Table 1- Composition and runtimes of simulated systems.

System	Lipid composition[a]	Solvent composition[b]	Number of replicates/windows	Minimum time per replicate/window (µs)[c]
Repulsive potential pore formation	*Cytoplasmic*<br>DPPC:21<br>MOPC:69<br>MLPC:10<br>POPC:106<br>PLPC:42<br>DOPC:44<br>PQPC:20<br>SUPC:19<br>POPE:57<br>PLPE:15<br>DOPE:49<br>SAPE:31<br>SUPE:30<br>POPG:12<br>DOPI:8<br>SQPI:5<br>SAPI:11<br>SOPS:12<br>SQPS:5<br>*Periplasmic*<br>DPPC:22<br>MOPC:73<br>MLPC:11<br>POPC:112<br>PLPC:44<br>DOPC:47<br>PQPC:22<br>SUPC:20<br>POPE:22<br>PLPE:6<br>DOPE:18<br>SAPE:12<br>SUPE:11<br>POPG:16<br>DOPI:24<br>SQPI:15<br>SAPI:35<br>SOPS:36<br>SQPS:15	W:28793<br>Na^+:414<br>CL^-:220	3	8.5
Umbrella-sampling pore formation	*Symmetric*<br>POPC:676	W:29606<br>Na^+:326<br>CL^-:326	45	0.1
	Symmetric<br>PAPC:676	W:29606<br>Na^+:326<br>CL^-:326	45	0.1

[a] Headgroup acronyms: PC: phosphocholine; PE: phosphoethanolamine; PG: phosphoglycerol; PI: phosphoinositol; PS: phosphoserine.
Lipid acronyms: DPPC: PC(di16:0 or 32:0); MOPC: PC(14:0-18:1 or 32:1); MLPC: PC(14:0-18:2 or 32:2); POPC: PC(16:0-18:1 or 34:1); PLPC: PC(16:0-18:2 or 34:2); DOPC: PC(di18:1 or 36:2); PQPC: PC(16:0-20:3 or 36:3); SUPC: PC(18:0-20:5 or 38:5); POPE: PE(16:0-18:1 or 34:1); PLPE: PE(16:0-18:2 or 34:2); DOPE: PE(di18:1 or 36:2); SAPE: PE(18:0-20:4 or 38:4); SUPE: PE(18:0-20:5 or 38:5); POPG: PG(16:0-18:1 or 34:1); DOPI: PI(di18:1 or 36:2); SQPI: PI(18:0-20:3 or 38:3); SAPI: PI(18:0-20:4 or 38:4); SOPS: PS(di18:1 or 36:1); SQPS: PS(18:0-20:3 or 38:3).
[b.] W counts refer to Martini waters, which correspond to 4 atomistic waters each.
[c] Total simulation time, including extended umbrella windows: 57.8 μs.

Supplementary Table 2: Molecular Dynamics simulations checklist

**Reliability and reproducibility checklist for molecular dynamics simulations** ***All boxes must be marked YES by acceptance unless an N/A option is available**	**Yes**	**N/A**	**Response (Please state where this information can be found in the text)**
**1. Convergence of simulations and analysis**			
1a. Is an evaluation presented in the text to show that the property being measured has equilibrated in the simulations (*e.g.* time-course analysis)?	☒		Methods, legend of Fig. 4 and SI
1b. Then, is it described in the text how simulations are split into equilibration and production runs and how much data were analyzed from production runs?	☒		Methods, legend of Fig. 4 and SI
1c. Are there at least 3 simulations per simulation condition with statistical analysis?	☒		Methods, legend of Fig. 4 and SI
1d. Is evidence provided in the text that the simulation results presented are independent of initial configuration?	☒		Methods, legend of Fig. 4 and SI
**2. Connection to experiments**			
2a. Are calculations provided that can connect to experiments (*e.g.* loss or gain in function from mutagenesis, binding assays, NMR chemical shifts, J-couplings, SAXS curves, interaction distances or FRET distances, structure factors, diffusion coefficients, bulk modulus and other mechanical properties, *etc.*)?	☒		Pg. 8, Figure 4
**3. Method choice**			
3a. Is it described in the text what force field and water model are used and why?	☒		Pg. 8, Methods
3b. Do simulations contain membranes, membrane proteins, intrinsically disordered proteins, glycans, nucleic acids, polymers, or cryptic ligand binding?	☒	☐	Pg. 8, Methods, legend of Fig. 4
If 3b is **YES**, are enhanced sampling methods used?	☒	☐	Methods
If enhanced sampling methods are used, are the convergence criteria clearly stated?	☒		SI
If 3b is **YES**, is it explained in the text why or why not enhanced sampling methods are used?	☒		Methods
**4. Code and reproducibility**			
4a. Is a table provided describing the system setup, such as simulation box dimensions, total number of atoms, total number of water molecules, salt concentration, lipid composition (number of molecules and type)?	☒		SI
4b. Is it described in the text what simulation and analysis software and which versions are used?	☒		Methods
4c. Are initial coordinate and simulation input files and a coordinate file of the final output provided as supplementary files or in a public repository?	☒		Data and Code availability
4d. Is there custom code or custom force field parameters?	☒	☐	Data and Code availability

	If **YES**, are they provided as supplementary profiles or in a public repository?	☒		Data and Code availability
