# Supplementary material for: Lipid unsaturation promotes BAX and BAK pore activity during apoptosis
Source: Nat Commun. 2024 Jun 3;15:4700. doi: 10.1038/s41467-024-49067-6 (PMC11148036; doi:10.1038/s41467-024-49067-6)
Supplement: Supplementary file 3 — Description of Additional Supplementary Files [file 41467_2024_49067_MOESM3_ESM.pdf]

Description of Additional Supplementary Files

File Name: Supplementary Data 1
Description: Cardiolipins metadata contains spectral data and associated data (Nano-ESI-MS/MS) used to identify metabolites relevant for hypothesis generation.

File Name: Supplementary Data 2
Description: Ceramides/sphingomyelins metadata contains spectral data and associated data (Nano-ESI-MS/MS) used to identify metabolites relevant for hypothesis generation.

File Name: Supplementary Data 3
Description: Fatty acids metadata contains spectral data and associated data (Nano-ESI-MS/MS) used to identify metabolites relevant for hypothesis generation.

File Name: Supplementary Data 4
Description: GPLs Metadata contains spectral data and associated data (Nano-ESI-MS/MS) used to identify metabolites relevant for hypothesis generation.
