# Supplementary material for: Lipid unsaturation promotes BAX and BAK pore activity during apoptosis
Source: Nat Commun. 2024 Jun 3;15:4700. doi: 10.1038/s41467-024-49067-6 (PMC11148036; doi:10.1038/s41467-024-49067-6)
Supplement: Supplementary file 8 — Reporting Summary [file 41467_2024_49067_MOESM8_ESM.pdf]

Corresponding author(s): Prof. Ana J. García-Sáez

Last updated by author(s): May 22, 2024

Reporting Summary

Nature Portfolio wishes to improve the reproducibility of the work that we publish. This form provides structure for consistency and transparency in reporting. For further information on Nature Portfolio policies, see our Editorial Policies and the Editorial Policy Checklist.

Statistics

For all statistical analyses, confirm that the following items are present in the figure legend, table legend, main text, or Methods section.

n/a	Confirmed	
☐	☒	The exact sample size (*n*) for each experimental group/condition, given as a discrete number and unit of measurement
☐	☒	A statement on whether measurements were taken from distinct samples or whether the same sample was measured repeatedly
☐	☒	The statistical test(s) used AND whether they are one- or two-sided<br>*Only common tests should be described solely by name; describe more complex techniques in the Methods section.*
☒	☐	A description of all covariates tested
☐	☒	A description of any assumptions or corrections, such as tests of normality and adjustment for multiple comparisons
☐	☒	A full description of the statistical parameters including central tendency (e.g. means) or other basic estimates (e.g. regression coefficient) AND variation (e.g. standard deviation) or associated estimates of uncertainty (e.g. confidence intervals)
☐	☒	For null hypothesis testing, the test statistic (e.g. *F*, *t*, *r*) with confidence intervals, effect sizes, degrees of freedom and *P* value noted<br>*Give P values as exact values whenever suitable.*
☒	☐	For Bayesian analysis, information on the choice of priors and Markov chain Monte Carlo settings
☒	☐	For hierarchical and complex designs, identification of the appropriate level for tests and full reporting of outcomes
☒	☐	Estimates of effect sizes (e.g. Cohen's *d*, Pearson's *r*), indicating how they were calculated

Our web collection on statistics for biologists contains articles on many of the points above.

Software and code

Policy information about availability of computer code

Data collection	Lipidomimcs data were acquired using Analyst 1.7.3 software (SCIEX), Simulation data was collected using the freely-available GROMACS suite, version 2021.
Data analysis	Lipidomics data were analyzed using LipidView 1.2 and MultiQuant 3.0.3 softwares (SCIEX). Percentage of GUVs internalizing the fluorescent probes were analyzed using GUVdetector software (https://doi.org/10.1093/bioinformatics/btu102), TMRE intensity analyzed using ImageJ/FIJI (https://doi.org/10.1038/nmeth.2019), all the statistical test were analyzed using GraphPad Prism 7.0 (https://www.graphpad.com/scientific-software/prism/). Simulation data analysis was carried out with the open-source MDAnalysis and NumPy Python packages. Confidence intervals and p-values for difference of means were computed using the also open-source scikits.bootstrap and SciPy Python packages. Specific code for calculating enrichment is available at https://www.itqb.unl.pt/labs/multiscale-modeling/downloads/Dadsena_etal2.tgz

For manuscripts utilizing custom algorithms or software that are central to the research but not yet described in published literature, software must be made available to editors and reviewers. We strongly encourage code deposition in a community repository (e.g. GitHub). See the Nature Portfolio guidelines for submitting code & software for further information.

Data

Policy information about availability of data

All manuscripts must include a data availability statement. This statement should provide the following information, where applicable:

- Accession codes, unique identifiers, or web links for publicly available datasets
- A description of any restrictions on data availability
- For clinical datasets or third party data, please ensure that the statement adheres to our policy

All data supporting the findings of this manuscript are available from the corresponding authors upon reasonable request.

Research involving human participants, their data, or biological material

Policy information about studies with human participants or human data. See also policy information about sex, gender (identity/presentation), and sexual orientation and race, ethnicity and racism.

Reporting on sex and gender	*Use the terms sex (biological attribute) and gender (shaped by social and cultural circumstances) carefully in order to avoid confusing both terms. Indicate if findings apply to only one sex or gender; describe whether sex and gender were considered in study design; whether sex and/or gender was determined based on self-reporting or assigned and methods used. Provide in the source data disaggregated sex and gender data, where this information has been collected, and if consent has been obtained for sharing of individual-level data; provide overall numbers in this Reporting Summary. Please state if this information has not been collected. Report sex- and gender-based analyses where performed, justify reasons for lack of sex- and gender-based analysis.*
Reporting on race, ethnicity, or other socially relevant groupings	*Please specify the socially constructed or socially relevant categorization variable(s) used in your manuscript and explain why they were used. Please note that such variables should not be used as proxies for other socially constructed/relevant variables (for example, race or ethnicity should not be used as a proxy for socioeconomic status). Provide clear definitions of the relevant terms used, how they were provided (by the participants/respondents, the researchers, or third parties), and the method(s) used to classify people into the different categories (e.g. self-report, census or administrative data, social media data, etc.) Please provide details about how you controlled for confounding variables in your analyses.*
Population characteristics	*Describe the covariate-relevant population characteristics of the human research participants (e.g. age, genotypic information, past and current diagnosis and treatment categories). If you filled out the behavioural & social sciences study design questions and have nothing to add here, write "See above."*
Recruitment	*Describe how participants were recruited. Outline any potential self-selection bias or other biases that may be present and how these are likely to impact results.*
Ethics oversight	*Identify the organization(s) that approved the study protocol.*

Note that full information on the approval of the study protocol must also be provided in the manuscript.

Field-specific reporting

Please select the one below that is the best fit for your research. If you are not sure, read the appropriate sections before making your selection.

☒ Life sciences ☐ Behavioural & social sciences ☐ Ecological, evolutionary & environmental sciences

For a reference copy of the document with all sections, see nature.com/documents/nr-reporting-summary-flat.pdf

Life sciences study design

All studies must disclose on these points even when the disclosure is negative.

Sample size	*Describe how sample size was determined, detailing any statistical methods used to predetermine sample size OR if no sample-size calculation was performed, describe how sample sizes were chosen and provide a rationale for why these sample sizes are sufficient.*
Data exclusions	no data were excluded
Replication	All cell experiments were performed measuring at least duplicates-triplicates during each individual experiment and means were calculated. These experiments were repeated independently at least three times to obtain at least three independent means. All figures show means calculated from these three means +/- SD. All experimental replicates successfully validated the experimental findings.
Randomization	*Describe how samples/organisms/participants were allocated into experimental groups. If allocation was not random, describe how covariates were controlled OR if this is not relevant to your study, explain why.*
Blinding	*Describe whether the investigators were blinded to group allocation during data collection and/or analysis. If blinding was not possible, describe why OR explain why blinding was not relevant to your study.*

Reporting for specific materials, systems and methods

We require information from authors about some types of materials, experimental systems and methods used in many studies. Here, indicate whether each material, system or method listed is relevant to your study. If you are not sure if a list item applies to your research, read the appropriate section before selecting a response.

Materials & experimental systems

n/a	Involved in the study
☐	☒ Antibodies
☐	☒ Eukaryotic cell lines
☒	☐ Palaeontology and archaeology
☒	☐ Animals and other organisms
☒	☐ Clinical data
☒	☐ Dual use research of concern
☒	☐ Plants

Methods

n/a	Involved in the study
☒	☐ ChIP-seq
☒	☐ Flow cytometry
☒	☐ MRI-based neuroimaging

Antibodies

Antibodies used

anti-BAK (Cell Signaling Technology, #5023, clone D2E11, Lot-4),
anti-VDAC1(Cell Signaling Technology, #4661, clone D73D12, Lot-11),
anti-Cytochrome C (BD Pharmingen, #556433, clone 7H8.2C12, Lot-1235765),
anti-FADS2 (Antibodies onlines, #ABIN5963439),
anti-STING (Cell Signaling Technology; #50494, clone D1V5L, Lot-1),
anti-IRF3 (Cell Signaling Technology T #4302, clone D83B9, Lot-7),
anti-Phospho-IRF-3 (Cell Signaling Technology; #29047, (Ser396), clone D6O1M, Lot-5),
anti-TBK1/NAK (Cell Signaling Technology, #3013, Lot-4),
anti-Phospho-TBK1 (Cell Signaling Technology, #5483, (S172), clone D52C2, Lot-13),
anti-Actin (Sigma; #A5316, clone AC-74, Lot-059M477DV),
peroxidase-conjugated goat anti-mouse (Jackson Immuno Research # 115-035-006, Lot- 131737),
peroxidase-conjugated goat anti-rabbit (Jackson Immuno Research, # 111-035-003, Lot-133599),
IRDye® 800CW Donkey anti-Mouse IgG Secondary Antibody (LI-COR, #926-32212),
IRDye® 680LT Donkey-anti-Rabbit Antibody (LI-COR, #926-68023).

Validation

Validation (for species; applications)
anti-BAK (H/M/R; WB, IP, IHC) has been validated by supplier.
anti-VDAC1(H/M/R/Mk; WB, IHC,) has been validated by supplier.
anti-Cytochrome C (H/M; WB,) has been validated by supplier.
anti-FADS2 (H/M; WB) has been validated by supplier.
anti-STING (H/M/R; WB, IP) has been validated by supplier.
anti-IRF3 (H/M/R/Mk; WB, IP) has been validated by supplier.
anti-Phospho-IRF-3 (H/M/R; WB, IP, IF, F) has been validated by supplier.
anti-TBK1/NAK (H/M/R/Mk; WB, IP) has been validated by supplier.
anti-Phospho-TBK1(H/M; WB, IP, IF, F) has been validated by supplier.
anti-Actin (H/M/R; WB, ELISA, IHC, IF) has been validated by supplier.
*Abbreviations: H-Human, M-Mouse, R-Rat, Mk-Monkey, WB-Western Blot, IP-Immunoprecipitation, IHC-Immunohistochemistry, IF-Immunofluorescence, F-Flow Cytometry

Eukaryotic cell lines

Policy information about cell lines and Sex and Gender in Research

Cell line source(s)

Human bone osteosarcoma cells: U2OS WT and U2OS Δ BAK, Gift from S. Tait, Glasgow
U2OS Δ BAK + mEGFP BAK were generated in the lab by G418 selection pressure
Mouse Embryonic fibroblast cells: MEF, Gift from M. Pasparakis, Cologne
Kidney epithelial cells (HK2), Clear cell Renal cancer cells (786-O, and OS-RC-2), Gift from C. Frezza, Cologne
Lung cancer cells (PC9, A549, H460, H358 and H1975), Gift from N. Peltzer, Cologne

Authentication

none of the cell line used were authenticated

Mycoplasma contamination

All cell lines used in this study were subjected to regular mycoplasma testing using kit (MycoStrip; Invivogen).

Commonly misidentified lines
(See ICLAC register)

none of the cell lines used are commonly misidentified lines.
